# Molecular Weight Distribution and Dissolution Behavior of Lignin in Alkaline Solutions

**DOI:** 10.3390/polym13234166

**Published:** 2021-11-28

**Authors:** Jie Yang, Mengya Sun, Liang Jiao, Hongqi Dai

**Affiliations:** Jiangsu Co-Innovation Center of Efficient Processing and Utilization of Forest Resources, Nanjing Forestry University, Nanjing 210037, China; yangxiaojie@njfu.edu.cn (J.Y.); sunmengyade@163.com (M.S.); johnallen_njfu@sina.com (L.J.)

**Keywords:** wood, alkali, lignin, molecular

## Abstract

Lignin, as the sole renewable aromatic resource in nature, has great potential for replacing fossil resources. However, the complexity of its structure limits its high value utilization, and the molecular weight distribution and dissolution behavior of lignin in alkaline solutions is still unclear. In this study, a conventional lignin separation during the pulping process in an alkaline hydrothermal system was performed by controlling the amount of NaOH, reaction temperature and holding time. Various analysis methods, including GPC, 2D–HSQC NMR and FTIR were used to study the characteristics of lignin fragments dissolved from wood. We were aiming to understand the rule of lignin dissolution and the recondensation mechanism during the process. The results showed dissolution of lignin due to ether bond fracturing by OH^−^ attacking the Cα or Cβ positions of the side chain with penetration of NaOH, and the lignin fragments in solution recondensed into complex lignin with more stable C–C bonds. The experimental results also prove that the average molecular weight increased from 4337 g/mol to 11,036 g/mol and that holding time from 60 min to 120 min at 150 °C with 14 wt% of NaOH.

## 1. Introduction

**Figure 1:**
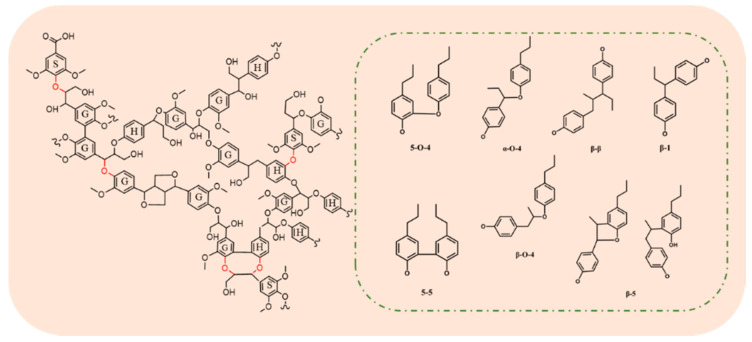
Structure of lignin and basic bond types between units.

Lignocellulosic biomass is recognized as a low-cost and easily available renewable resource. It is mainly composed of cellulose, hemicellulose and lignin [1]. Among these three components, lignin generally accounts for 15–30 wt% of dry biomass, and is composed of three basic structural units—guaiacyl propane, syringyl propane and p-hydroxyphenyl propane—randomly linked by C–O and C–C bonds (Figure 1). Due to its unique structure, lignin is considered to be the sole renewable aromatic resource in nature [2,3,4,5]. The efficient conversion of lignin to fuels and chemicals has thus been recognized as the most promising way to substitute fossil resources. For example, some aromatic compounds obtained from lignin have great application potential in the fields of materials [6,7], catalysts [8], biological science [9], chemical energy [10] and others. However, the complexity of lignin’s amorphous structure significantly hinders its high value-added utilization.

Among the various C–C and C–O linkages mentioned above, the β–O–4 bond is the most abundant (40–80%) and also most prone to fracturing [11,12,13,14]. Reducing the average molecular weight of lignin by breaking the ether bonds, particularly the β–O–4 bond, can greatly improve its application potential. Based on previous publications, although many of methods for lignin degradation have been developed, such as oxidation degradation [15,16], microbial degradation [17,18], catalytic degradation [19] and hydrogenation reduction degradation [20,21,22,23], most of them have to be completed with the help of harsh experimental conditions and catalysts, due to the inevitable recondensation of lignin fragments. Moreover, most studies on the degradation mechanism were conducted using model lignin compounds, which cannot truly represent the real degradation mechanism of lignin [24].

**Figure 2:**
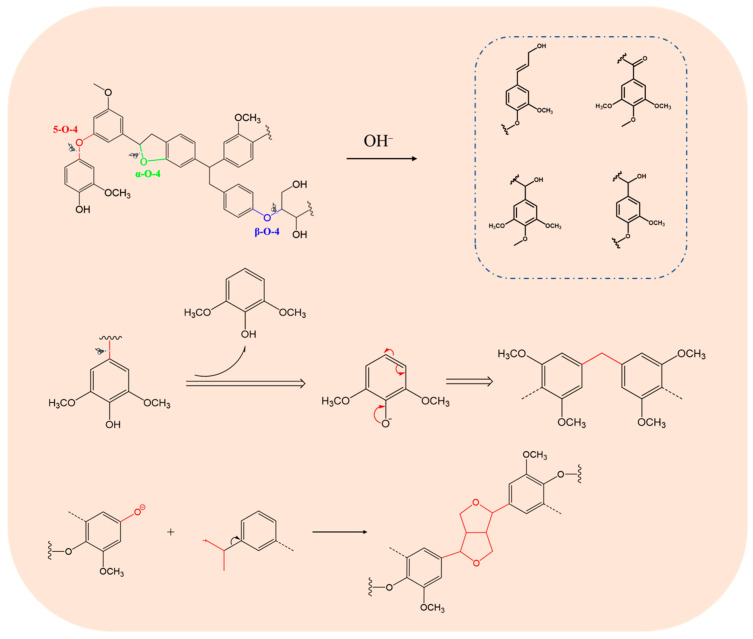
Reported mechanisms of lignin degradation and polymerization.

Interestingly, in the pulping process of the paper industry, large amounts of fragments lignin are produced. On the one hand, the ether bonds can be broken due to the chemical reactions between OH– as a nucleophile and phenolic hydroxyl groups in lignin, which lead to the degradation of lignin macromolecules into alkali lignin with smaller molecular weight. In addition, strong alkali can also break the ester bonds between lignin and holocellulose, thereby accelerating the separation of lignin [25,26]. On the other hand, the lignin fragments are easily recondensed with each other during the continuous degradation and dissolution of lignin. These two behaviors exist in an alkaline hydrothermal environment simultaneously. A schematic diagram of the lignin dissolution mechanism is shown in Figure 2 [27]. 

As we know, lignin is often discarded as waste in black liquor due to its complex structure and poor recovery [28]. The recondensation of lignin fragments during pulping increases the molecular weight of the final lignin, which makes it difficult to separate and purify for downstream applications. So far, the relationship between the average molecular weight distribution of lignin and pulping conditions is still unclear. In-depth study of this relationship could help us to obtain lignin of various molecular weights by controlling the process conditions or employing some polymerization inhibitor (e.g., methanol, methanal, etc.), thereby providing a theoretical basis for further degradation and conversion of lignin.

This study used wood flour as a raw material, and carefully investigated the effects of the pulping conditions (e.g., the reaction temperature, holding time and dosage of NaOH) on the dissolution and condensation behavior of alkaline lignin in a hydrothermal system. After adjusting the pH to precipitate lignin, the molecular weight distribution and typical structural characteristics were analyzed in detail. Finally, the relationships between the pulping conditions and the molecular weight distribution of lignin are preliminarily illustrated, and the optimal pulping conditions toward lowest lignin molecular weight were obtained.

## 2. Materials and Methods

### 2.1. Feedstock and Chemicals

Eucalyptus (hardwood) chips were obtained from Nine Dragons Paper. A cutting mill was utilized to mill wood chips into powder with grains around 0.712 mm in diameter. Sodium hydroxide (NaOH), sulfuric acid (H_2_SO_4_), hydrochloric acid (HCl) and tetrahydrofuran (THF) were purchased from Nanjing Chemical Reagent Co., Ltd., Nanjing, China.

The composition analysis of raw biomass was performed according to the NREL [29] protocol. Results are as shown in Table 1:

### 2.2. Preparation of Alkali Lignin

The reaction was carried out in a 200 mL autoclave. The reaction temperatures were set to 110, 120, 130, 140, 150, 160 and 170 °C; and the amounts of NaOH were 12, 14, 16, 18, 20 and 22 wt%, respectively. After the temperature reached a set value, the autoclave was held at that temperature for 0, 10, 20, 30, 40, 50, 60, 90 or 120 min. At the end of the reaction, the reactor was cooled to ambient temperature using water-flow. The reaction mixture was filtered through qualitative filter paper, and washed with deionized water to neutral.

### 2.3. Products Separation

The solid residue obtained was dried at 105 °C for 4 h and weighed. We adjusted the pH of the filtrate to 2 at 65 °C with 0.2 mol/L H_2_SO_4_, centrifuged the solution at 12 °C for 10 min and collect the precipitates to obtain the alkali lignin.

### 2.4. Product Analysis

#### 2.4.1. Composition Analysis of Raw Materials

The moisture content of the wood powder was determined by calculating the mass loss after drying the wood flour in an oven at 105 °C for 4 h. The chemical composition of wood powder was tested by the two-step hydrolysis method of concentrated acid and dilute acid. The specific operation was as follows: 0.3 g wood powder was put into a 100 mL bottle and treated with 72% H_2_SO_4_ for 60 min, stirring every 5–10 min during the process. Deionized water was added—mixture diluted to 4%; we put it into an autoclave; it reacted at 121 °C for 1 h. We filtered the residue with a G3 glassy hopper, took 50 mL of filtrate to measure the contents of acid-soluble lignin, cellulose and hemicellulose, dried the filtrate at 105 ± 3 °C, weighed and recorded the results. The mixture was transferred to a muffle furnace after drying. Ash content was measured after burning at 575 ± 25 °C for 4 h.

#### 2.4.2. Analysis of FT-IR

A certain amount of lignin and potassium bromide were mixed and ground in an agate mortar for tablet pressing. An FT-IR-650 (Tianjin Port East Technology Development Co., Ltd., Tianjin, China) Fourier transform infrared spectrometer was used. The scanning range was 400–4000 cm^−1^ and the scanning was iterated 32 times.

#### 2.4.3. Analysis of GPC

Before the determination by gel permeation chromatography (GPC), the acetylated lignin was pretreated to make it soluble in tetrahydrofuran (THF): 0.1 g of alkali lignin was dissolved in a 2 mL pyridine–acetic anhydride mixture and reacted at room temperature for 72 h. The sample was washed with 1% HCl, and the acetylation lignin was obtained by centrifugation. Acetylated lignin was dissolved in THF at 1 mg/mL. The relative molecular weight of acetylated lignin was determined by GPC (Shimadzu Class LC-10 system, Shimadzu, Japan). The GPC analysis was conducted at 40 °C using THF as the mobile phase with a flow rate of 1 mL/min. 

#### 2.4.4. Analysis of SEM

Scanning electron microscopy (SEM) (Quanta-200, FEI, Shelbyville, KY, USA) was performed to observe the surface morphology of the pulps after different holding times.

#### 2.4.5. Analysis of NMR

The structures of lignin samples during different holding times were analyzed by 2D^13^C-^1^H heteronuclear single quantum correlation (HSQC) NMR. Generally, 50 mg samples were dissolved in 0.5 mL of DMSO-d6 for NMR analysis. NMR spectra were acquired for a total acquisition time of 12 h with a 14 ppm sweep width in the F2(^1^H) and 200 ppm sweep width in the F1 (^13^C).

## 3. Results

### 3.1. The Morphologies and Yields of the Residual Lignin and Pulp

**Figure 3:**
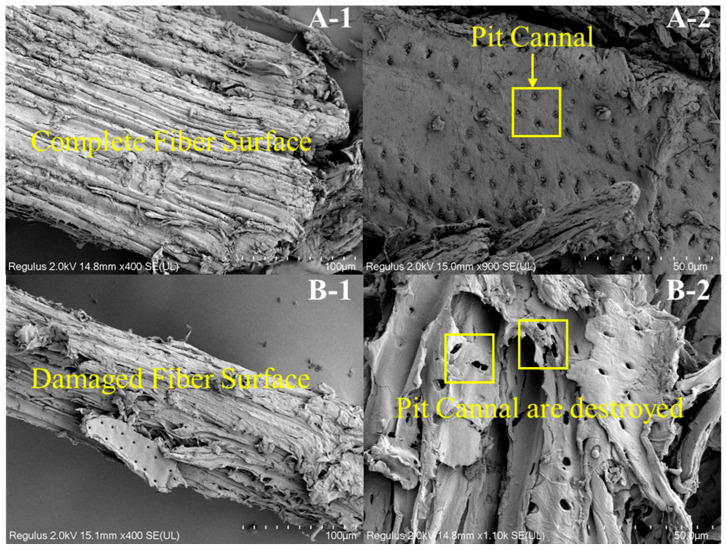
Photos of pulp at holding times 0 min (**A**) and 60 min (**B**). The specific scale bars with 100 μm (1) and 50 μm (2) are shown in the SEM images.

**Figure 4:**
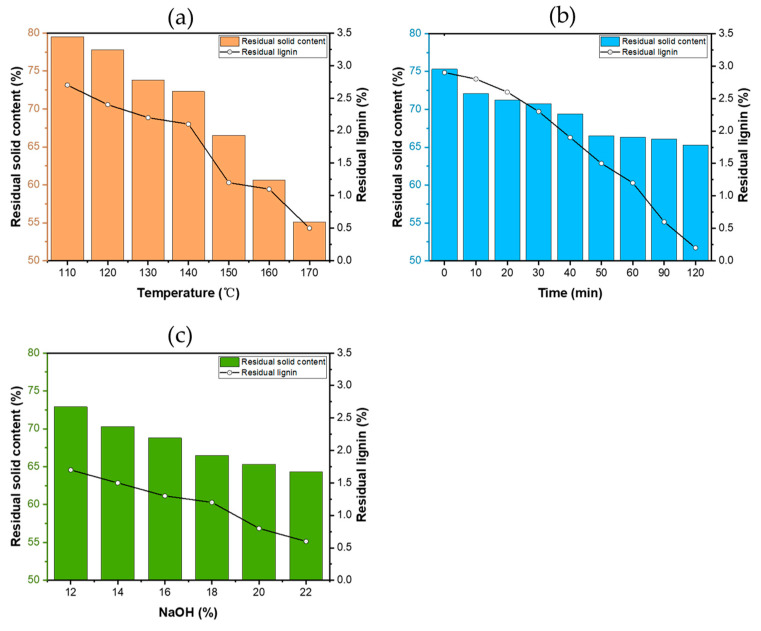
Yields of residual lignin and pulp under different reaction temperature (**a**), holding time (**b**) and dosage of NaOH (**c**).

The morphologies of the pulps obtained after different holding times can be seen in photographs and SEM images (Figure 3). A clear distinction could be observed among the pulp samples according to holding time. It is shown that cellulose fibers were tightly bound together through the cross-linking of lignin and hemicellulose in Figure 3. With the extension of holding time, the pit canals on the fibers were gradually destroyed in the pulp. Figure 3 (high magnification image) shows that the pit chambers on the fibers’ surfaces were destroyed by lye, indicating that the extension of holding time accelerated the dissolution of lignin. The contents of pulp and residual lignin are shown in Figure 4. As the reaction temperature increased, both the yield of pulp and the content of residual lignin decreased gradually, indicating that temperature is conducive to the dissolution of lignin; the composition of the pulp was still unstable and could be further deconstructed. With the extension of holding time, pulp content and residual lignin content gradually decreased; and no significant changes were found between samples once the time surpassed 60 min, indicating that a longer time is conducive to the infiltration of lye before 60 min. As NaOH dosage increased, the proportion of pulp and residual lignin content decreased gradually. The results confirmed that cellulose, hemicellulose and lignin were effectively separated by alkali during the whole reaction.

### 3.2. The Molecular Weight Distribution of Lignin

**Figure 5:**
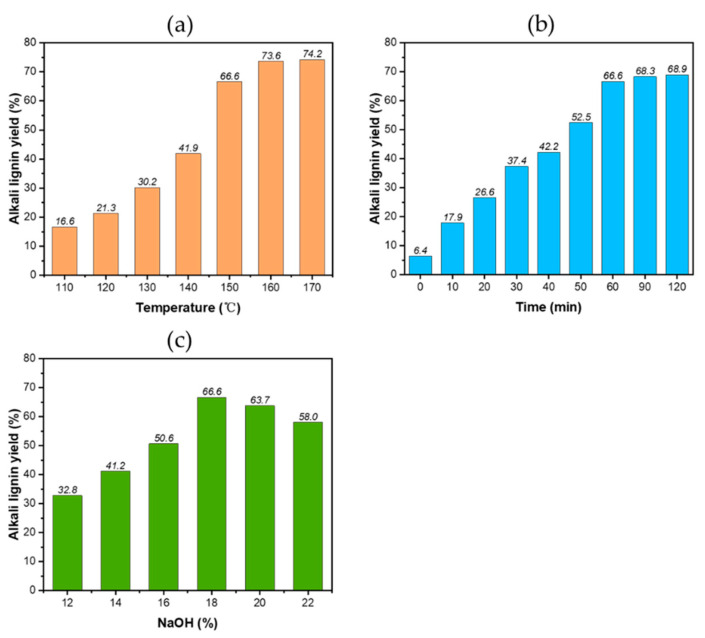
Yields of alkali lignin under different reaction temperature (**a**), holding time (**b**) and dosage of NaOH (**c**).

Figure 5 represents the changes of lignin yield under different reaction conditions. It was found that the lignin yield rapidly increased as the reaction temperature increased from 110 to 150 °C, at which temperatures, large amounts of lignin were dissolved. The lignin yield was stabile from 150 to 170 °C, caused by the dissolution of the residual lignin. As with the pulp yield shown in Figure 4, it was found that lignin was mainly dissolved in large quantities before 150 °C, and the fibers were not damaged below 150 °C. As the temperature continued to rise, the dissolution of cellulose rather than lignin occurred. With the extension of the holding time, the yield of lignin gradually increased, indicating that lignin is constantly dissolved in the whole reaction process with the continuous infiltration of lye. Holding for longer than 60 min plays an insignificant role in the dissolution of lignin. As NaOH dosage increased, lignin yield increased to begin with, and then decreased. The maximum yield was 66.6 wt% at a NaOH dosage of 18%. It is reported that some bonds between lignin and carbohydrate are sensitive to alkali and can be cleaved by alkali, leading to a significant decrease in pulp yield (Figure 4) and a large increase in lignin recovered from the solution. However, as the dosage of NaOH continued to increase from 18%, the lignin yield decreased slowly, which was caused by the degradation of a large amount of hemicellulose due to the NaOH, leading to more carbohydrates dissolving and then combining with lignin.

**Figure 6:**
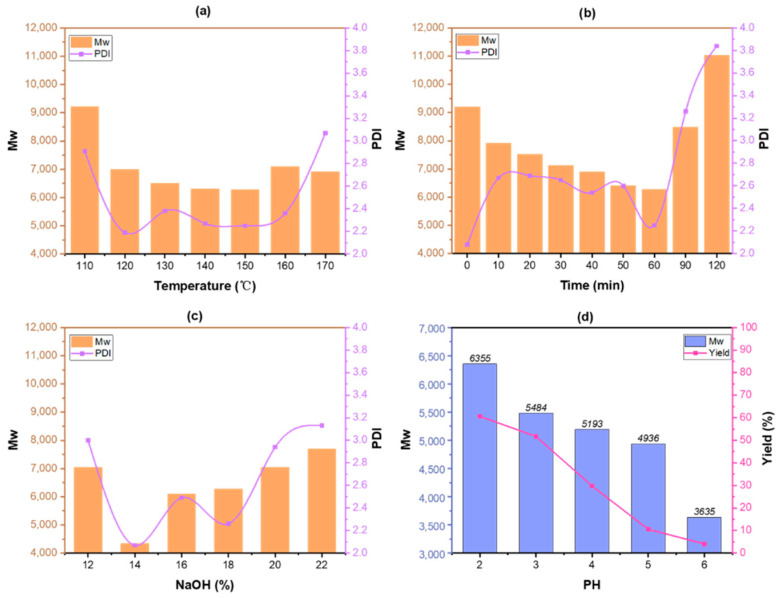
Distributions of average molecular weight (Mw) and polydispersity (PDI) of alkali lignin with different reaction temperatures (**a**), holding time (**b**) and dosage of NaOH (**c**), and the yield of lignin under different pH of acid precipitate (**d**).

Alkali lignin was prepared with different reaction temperatures, holding times and NaOH dosages. As can be seen from Figure 6, the average molecular weight of lignin was 4337 g/mol when the holding time was 60 min, reaction temperature was 150 °C and NaOH dosage was 14%. As the reaction process went on, lignin was dissolved from biomass under the action of strong alkali, and the molecular weight decreased continuously. However, when the holding time was longer than 60 min, it was found that the molecular weight increased, indicating that the recondensation reaction rate of lignin fragments had exceeded the dissolution rate of lignin after 60 min. In the process of acid separation of lignin, we adjusted the pH to 6, 5, 4, 3 or 2. When comparing the molecular weights of the lignin obtained under different pH conditions, it can be seen that the average molecular weight of lignin increased with the rise in pH value, indicating that lignin with higher molecular weight would be preferentially deposited.

### 3.3. The Structural Change of Lignin

**Figure 7:**
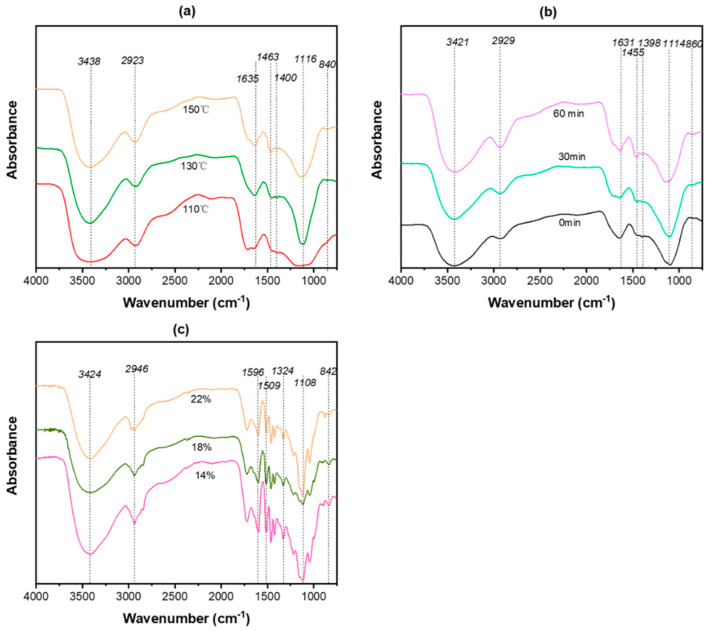
FT-IR spectra of alkali lignin under different reaction temperatures (**a**), holding times (**b**) and amounts of NaOH (**c**).

The FT-IR spectra of alkali lignin under different reaction conditions are shown in Figure 7. Each product contained a characteristic 1370–1445 cm^−1^ lignin absorption peak; see Figure 7a–c. The stretching vibration peak of O–H of the aromatic ring at 3600–3200 cm^−1^ increased with the reaction temperature and holding time, indicating that lignin fragmentation was intensified. The absorption peak at 2950–2848 cm^−1^ was attributed to the C–H methyl and methylene stretching vibrations of lignin. These results indicate that lignin fragments retained intact side chains, which was also the main reason for recondensation of lignin. The absorption peak at 1600–1510 cm^−1^ was assigned to benzene, which increased gradually with the rise of reaction temperature and holding time, indicating that the lignin fragment content was improved and the benzene ring structure of the products was not destroyed in the process of reaction. The absorption peaks at 1463 cm^−1^ (a), 1455 cm^−1^ (b) and 1509 cm^−1^ (c) correspond to C–H deformation and planes of –CH_2_ and –CH_3_ bending vibrations, and the peaks’ intensities increased gradually with temperature and time, which showed that lignin was broken and separated gradually as the reaction went on. However, the effect of the amount of NaOH on the structure of lignin was not obvious, which we can see in Figure 7c, suggesting that the addition of alkali mainly accelerated the removal rate of lignin, not the cross-linking of lignin fragments.

**Figure 8:**
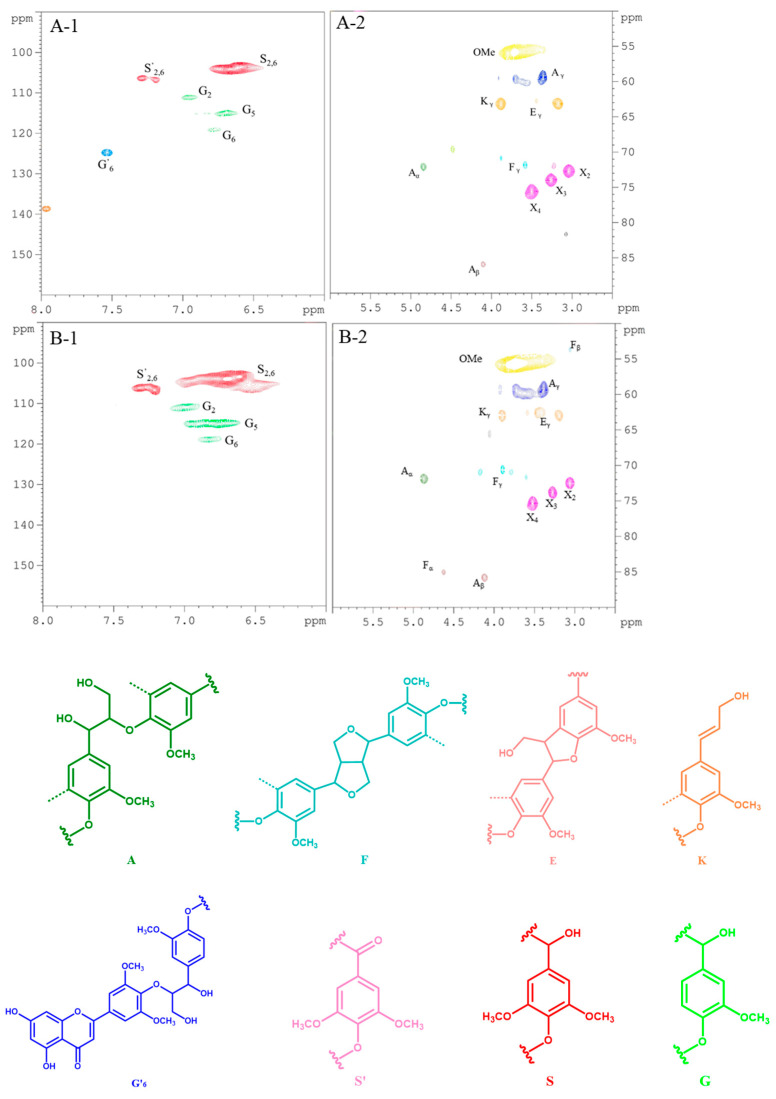
2–D HSQC NMR and corresponding structural analysis with aromatic region (1) and aliphatic region (2) of lignin at T150A14t0 (**A**) and T150A14t60 (**B**).

Figure 8 shows the HSQC spectra for alkali lignin with different of holding times. In addition in the figure, the structures of lignin can be observed and the structural changes of lignin can be compared [29,30,31,32]. The β–O–4 linkage is the most important linkage between lignin units, which can be observed at δC/δH 69.42/4.85 ppm, δC/δH 85.1/4.18 ppm and δC/δH 59.8/3.18–3.62 ppm, representing Cα–Hα, Cβ–Hβ and Cγ–Hγ, respectively. It can be seen that the signals of various bonds became stronger with the prolongation of the holding time, indicating that lignin was dissolved even in the last time period measured, and the proportions of β–O–4 and α–O–4 bonds increased with the rise in molecular weight. In addition, the phenylcoumarin (E) structure, and methoxyl (OMe, –OCH_3_) and Cγ positions on the p-hydroxycinnamol (K) structure were derived from δC/δH 62.5/3.71, δC/δH 55.2/3.74 and δC/δH 63.3/4.06 ppm. 

The signals of Cα–Hα at δC/δH 83.0/4.84 ppm, Cβ–Hβ at δC/δH 53.3/3.06 ppm and Cγ–Hγ at δC/δH 70.6/3.75–4.14 ppm increased in intensity with the extension of holding time. The signal of the β–O–4 bond was also enhanced. The peak intensities of S_2_ at δC/δH 103.9/6.7 ppm, G_2_ at δC/δH 111.0/7.07 ppm, G_5_ at δC/δH 114.5/6.7 ppm and G_6_ at δC/δH 119.0/6.78 ppm increased, indicating the continued dissolution of lignin with the extension of the holding time. At the same time, the G-type β–O–4 bond (G’_6_) at δC/δH 123.8/7.51 ppm disappeared, which was the main bond of lignin polymerization. This indicates that the dissolution and fracture rate of lignin before 60 min was faster than the rate of recondensation forming macromolecule lignin, which was the basis for our decision to adopt the holding time of one hour in the follow-up study.

## 4. Conclusions

The high value utilization of lignin is very difficult, and knowing the mechanism of lignin degradation is one of the bottlenecks in the process of optimizing lignin degradation and separation, which is worth further study. In this study, lignin separation was performed in a traditional alkaline hydrothermal system, and the structural changes of lignin dissolution were investigated. We found that the lignin fragmentation and polycondensation process are conducted at the same time, before 60 min; the dissolution and fracture rate of lignin was faster than that of lignin polycondensation; and as time went on, a large amount of polycondensation of lignin occurred. The polycondensation continued to increase the molecular weight of the lignin. Therefore, how to hinder the recondensation of lignin will be the key to high value utilization of it. At the same time, the low molecular weight lignin with Mw of 4337 can be prepared under the conditions of 150 °C, 60 min and 14 wt% NaOH, which will serve as the basis for subsequent research.

## Figures and Tables

**Table 1:** Contents of cellulose, lignin and hemicellulose in eucalyptus via NREL protocol.

Material	Holocellulose (%)	Acid-Insoluble Lignin (%)	Acid Soluble Lignin (%)	Ash Content (%)
Eucalyptus	71.54	19.60	4.71	0.06

Content of cellulose, lignin and hemicellulose in eucalyptus.

## Data Availability

The data presented in this study can be made public.
